# Supplementary material for: KPB82 potently and selectively inhibits ACER2 and protects tumor and primary cells from doxorubicin-induced cytotoxicity
Source: bioRxiv. 2026 Oct 2:2026.09.29.754712. Preprint. [Version 1] doi: 10.64898/2026.09.29.754712 (PMC13648641; doi:10.64898/2026.09.29.754712)
Supplement: Supplementary file 1 [file media-1.pdf]

Supplementary table S1. List of ceramidase inhibitors

Compound	IC_{50} value	Target
C6 Urea Ceramide	NA	Neutral ceramidase
D-erythro-MAPP	1-5 µM	Ceramidase
AD-2646	40 µM	Ceramidase
Ceranib1	NA	Ceramidase
Carmofur	29 nM	Acid ceramidase
Acid Ceramidase-IN-1	0.166 µM	Acid ceramidase
Acid Ceramidase-IN-2	NA	Acid ceramidase
ARN14974	79 nM	Acid ceramidase
ARN14988	12.8 nM	Acid ceramidase
SOBRAC	NA	Acid ceramidase
SACLAC	NA	Acid ceramidase
W000113414_I13	6.6µM	Acid ceramidase

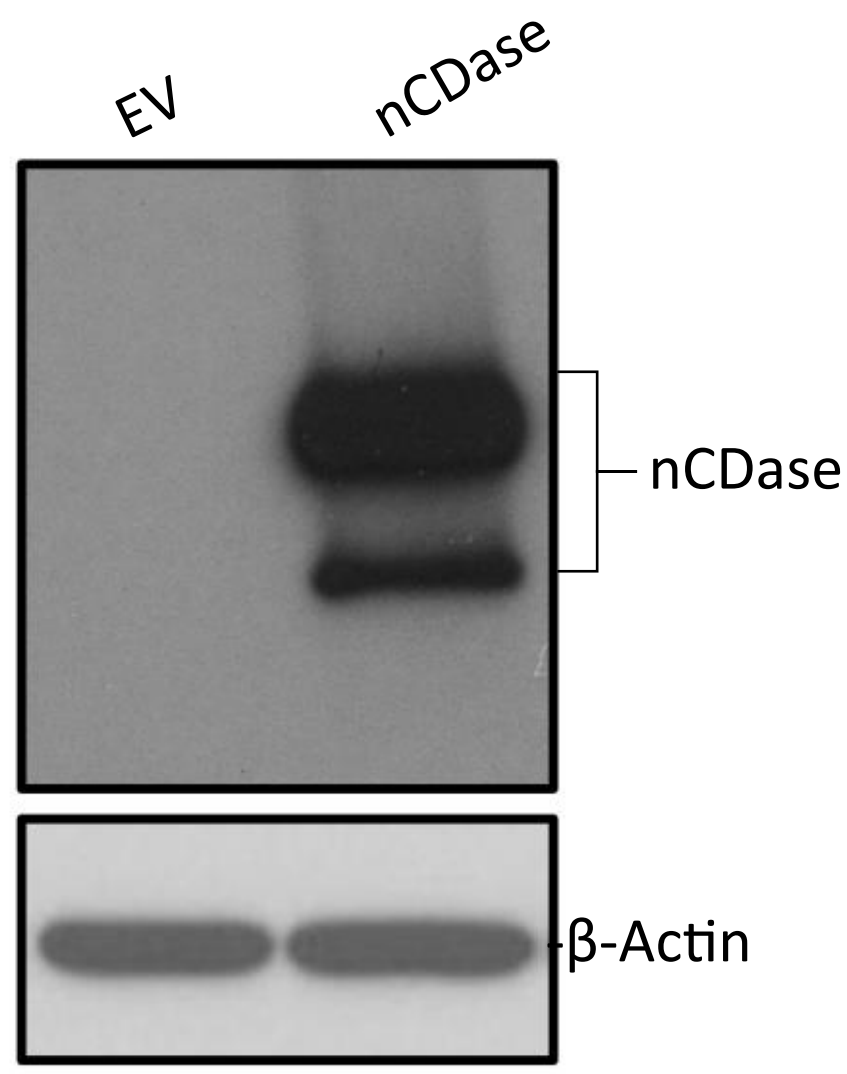

Figure S1. Neutral ceramidase overexpression in MCF7-ASAH2 cells

MCF7 cells, stably transfected with an ASAH2-expression construct or empty vector, were cultured as described in the Materials and Methods section. Total cell lysates were prepared from these cells and subjected to western blot analysis using anti-V5 peptide tag antibody.

Compound ID	3D representation	2D representation
LCL-847 (KPB82)	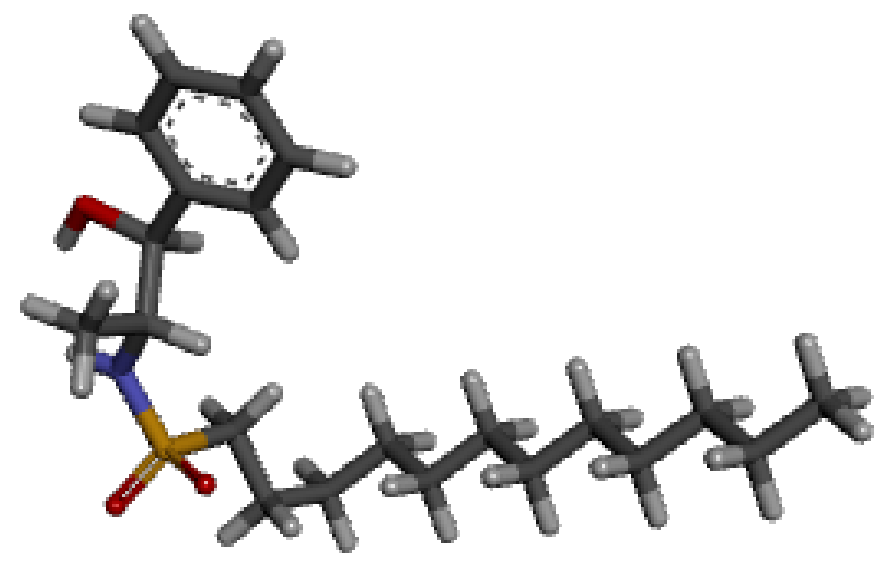	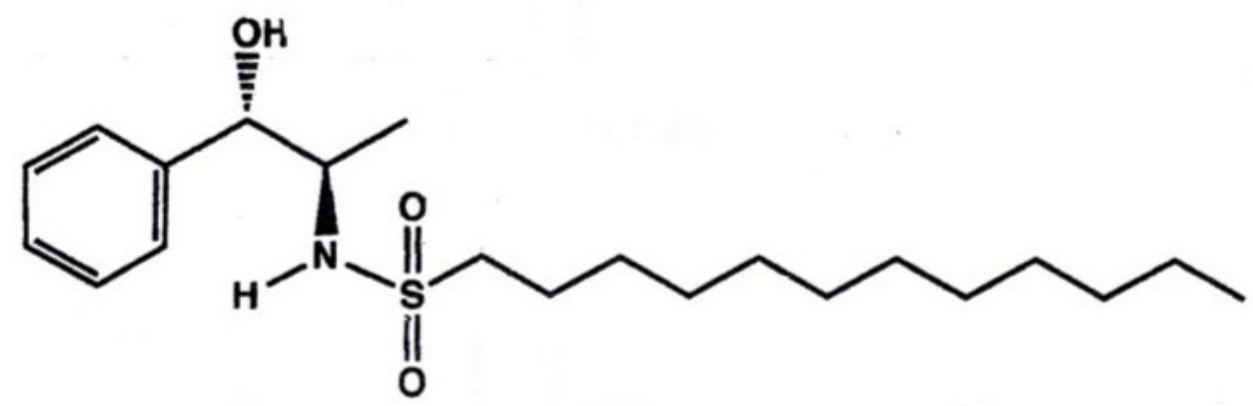
LCL-851	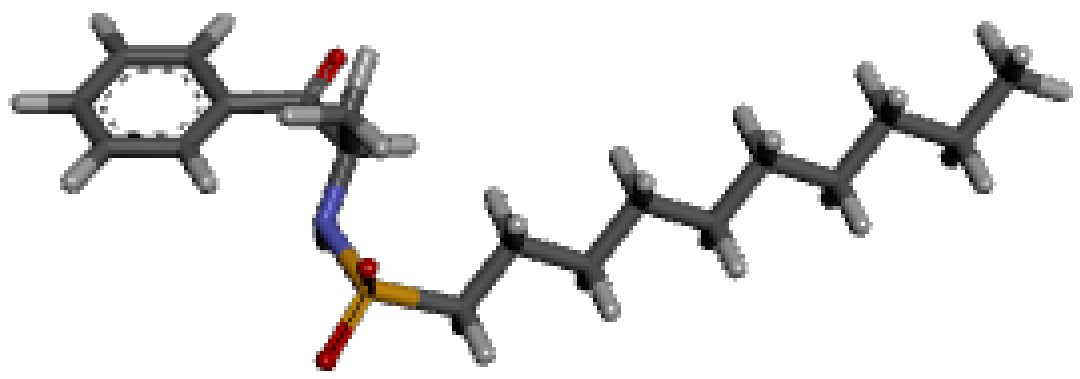	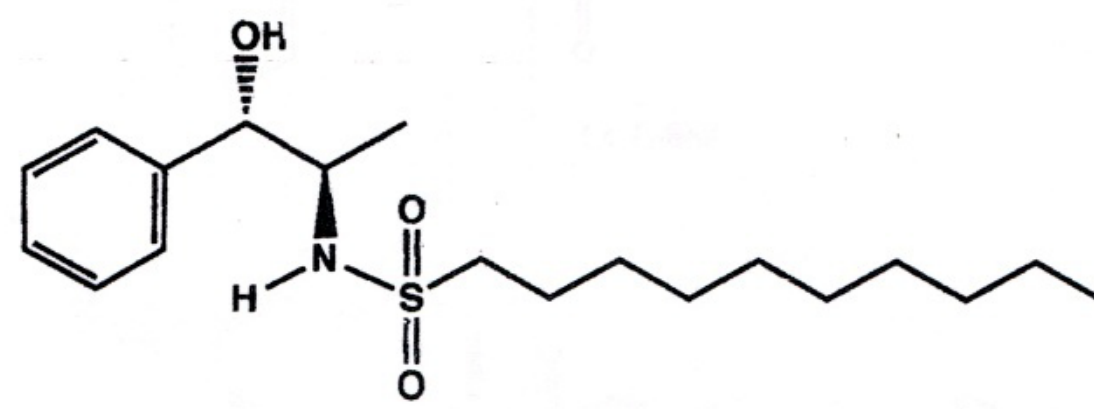
LCL-852	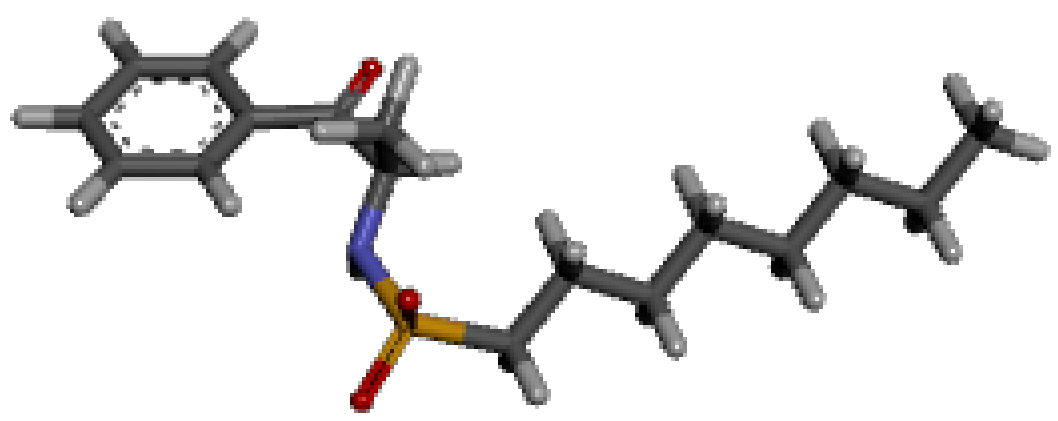	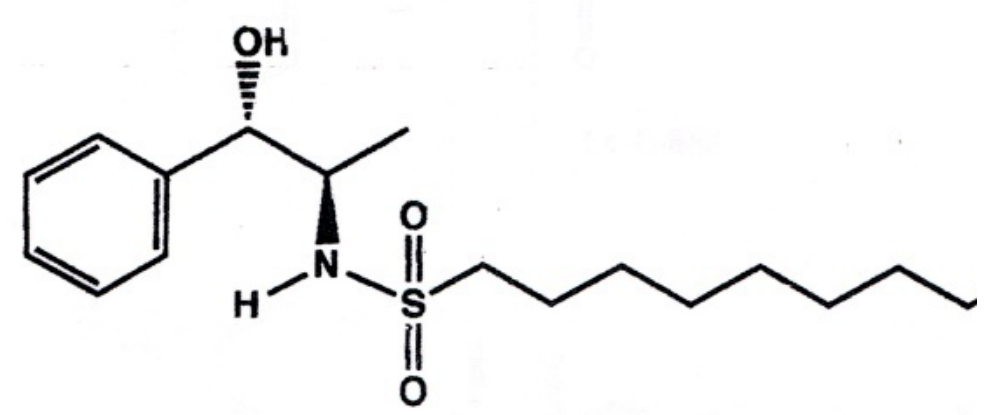
LCL-853	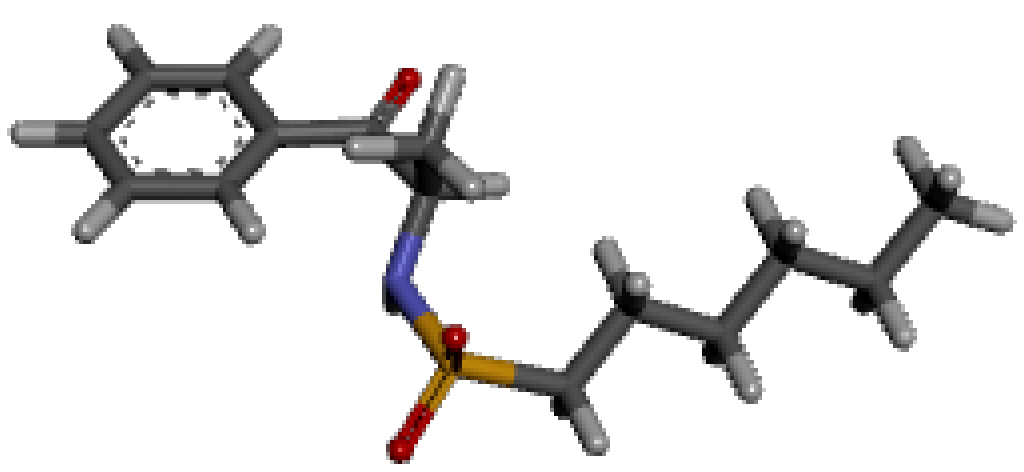	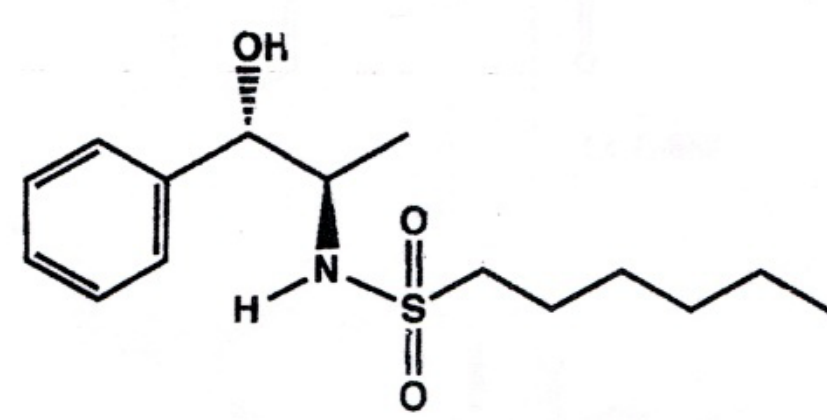

Figure S2. Chemical structures of short-chain analogs of KPB82

The chemical structures of KPB82 analogs with a 12-carbon (LCL-847), 10-carbon (LCL-851), 8-carbon (LCL-852), and 6-carbon (LCL-853) chains, respectively.

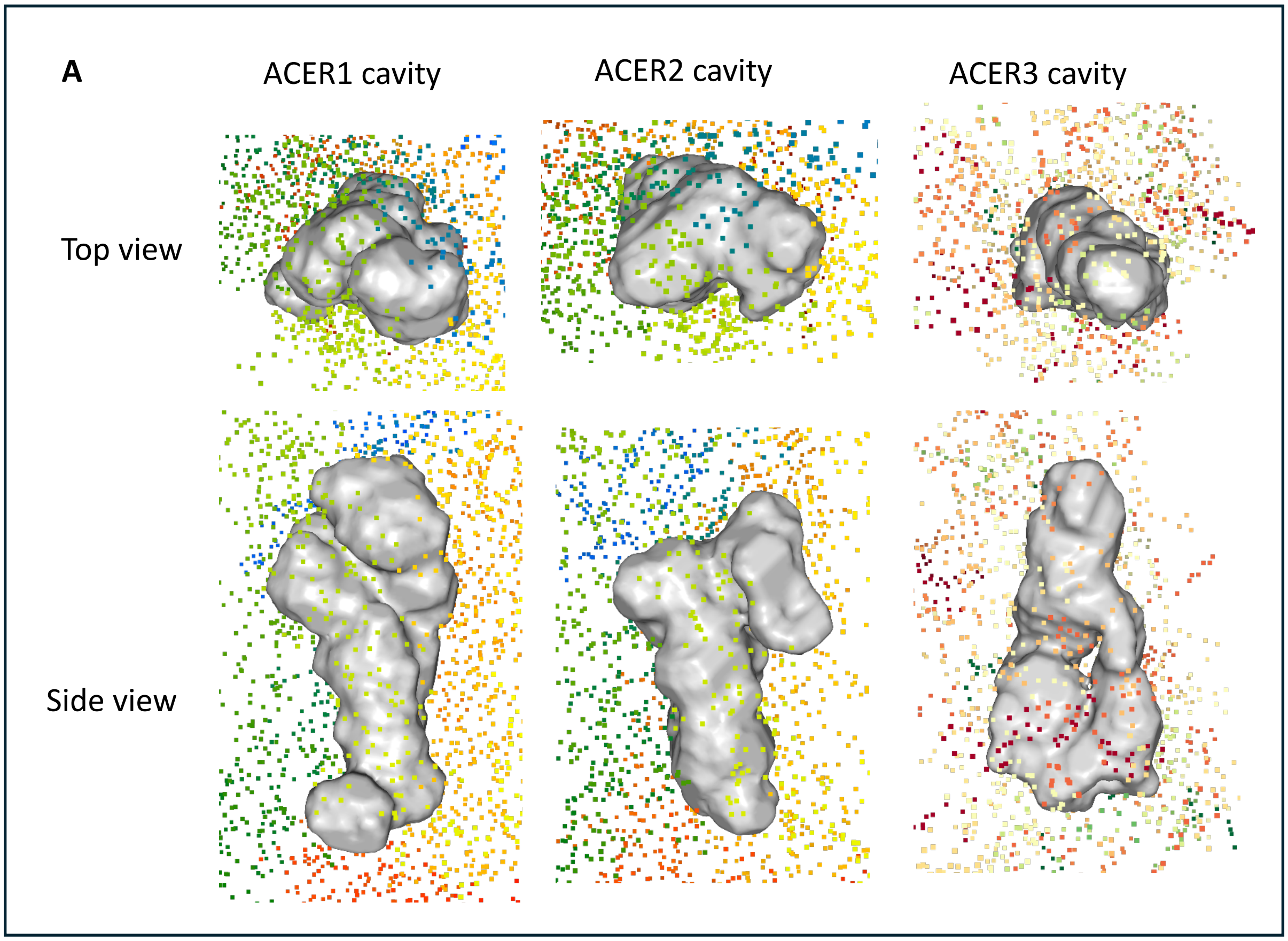

B	Area (Å²)	Vol. (Å³)	Avg Dep. (Å)	Max Dep. (Å)	Avg Hyd.
ACER1	1011.86	1210.46	15.88	29.01	-0.72
ACER2	762.11	1003.54	8.35	16.94	-0.65
ACER3	615	715.18	2.61	7.85	-0.38

Figure S3. Distinct architectures of substrate-binding pockets in the alkaline ceramidase family

The online software KVFinder was employed to predict cavities in the alkaline ceramidases. The largest central hydrophobic cavity from each ACER enzyme was shown (A) and their depths and volumes were listed (B).
